# The impact of the COVID-19 pandemic on eating disorder symptoms and COVID-19-related stress

**DOI:** 10.1186/s40337-026-01578-x

**Published:** 2026-03-27

**Authors:** Valencia van Heumen, Joyce Maas, Pia Burger, Mladena Simeunovic-Ostojic

**Affiliations:** 1 Department of Medical and Clinical Psychology, Tilburg University, Tilburg, The Netherlands; 2 Center of Eating Disorders, GGZ Oost-Brabant, Wesselmanlaan 25a, 5707 Helmond, HA The Netherlands

**Keywords:** COVID-19, Eating disorders, Stress, Anorexia nervosa, Bulimia nervosa

## Abstract

**Introduction:**

The coronavirus (COVID-19) has led to a worldwide pandemic with various consequences, such as heightened stress and an increase in the risk of developing psychological problems. The pandemic posed specific challenges for patients with eating disorders (ED), with worsening of ED symptoms as a possible consequence. This longitudinal prospective study investigated the associations between COVID-19-related stress and ED symptoms during periods of lockdown compared to reopening periods during the COVID-19 pandemic.

**Methods:**

Over a 2-year period, starting in August 2020, patients (N = 108) in specialized treatment for an ED completed monthly measurements of the Eating Disorder Examination Questionnaire (EDE-Q) and the COVID-19 Peritraumatic Distress Index (CPDI). Using mixed linear models, we investigated whether (1) ED symptoms were higher during lockdown periods compared to reopening periods, (2) COVID-19-related stress was higher during lockdown period when compared to reopening periods, (3) COVID-19-related stress was associated with higher levels of ED symptoms, and whether (4) COVID-19-related stress mediated the association between period (lockdown versus reopening) and ED symptoms.

**Results:**

Results showed a significant negative total effect between period and ED symptoms, with ED symptoms being lower during the reopening periods compared to the lockdown periods. Secondly, during lockdown periods, patients experienced more COVID-19-related stress than during the reopening periods. Thirdly, there was a significant positive association between COVID-19-related stress and ED symptoms. Finally, results showed a significant negative direct effect of period on ED symptoms, where, controlling for COVID-19-related stress, patients experienced fewer ED symptoms during the reopening periods compared to the lockdown periods (partial mediation).

**Conclusion:**

Both COVID-19-related stress and ED symptoms were higher during lockdown than reopening periods, and greater COVID-19-related stress was associated with more severe ED symptoms. The mediation analysis showed that ED symptoms were higher during lockdown than during reopening periods, partly due to more COVID-19-related stress during lockdown periods.

## Plain Language Abstract

This study examined the impact of the COVID-19 pandemic on individuals with eating disorders. Over a two-year period, 108 patients in specialized treatment completed monthly assessments of their eating disorder symptoms and COVID-19-related stress. Results indicated that both stress and eating disorder symptoms were higher during lockdown periods compared to periods when restrictions were lifted. Furthermore, COVID-19-related stress partly explained the increase in eating disorder symptoms during lockdown, highlighting the important role of stress in exacerbating symptoms.

## Introduction

The COVID-19 pandemic and the ensuing measures have profoundly affected mental health worldwide. Initial research indicated increased levels of stress and depression in the general population during the pandemic [1–8]. This posed a specific risk to individuals with pre-existing mental health issues [9], as well as individuals with eating disorders (ED). For instance, the shift towards virtual socializing during the pandemic led to increased screen time, increasing preoccupation with their body image [10, 11]. Additionally, patients with an ED may be more exposed to online content concerned with eating, shape and weight [10, 12–14], which may exacerbate disordered eating behaviors and body image concerns. Several reviews and meta-analyses have been published during the COVID-19 pandemic [15–23], consistently showing deterioration of ED symptoms and general mental health as a result of the pandemic, including anxiety, depression, and suicidal ideation, among individuals suffering from EDs. There was an increased demand for specialist ED services, and a heightened risk for the onset of an ED.

Particularly vulnerable were children and adolescents, as well as adults with a current or a history of an ED, who showed a susceptibility to the pandemic’s negative effects and a propensity for relapse [24–26]. For instance, Meier et al. [21] reported a significant overall increase in incidence (15%), hospital admissions (48%), and emergency department visits (11%) for patients with EDs during the pandemic.

While Schneider et al. [23] acknowledged a general worsening of symptoms, they also mentioned that not all individuals experienced a negative impact; some reported symptom improvement or stability. Gao et al. [16] particularly noted this for patients with anorexia nervosa, who may have benefited from fewer social stressors, stable family dynamics, and continued e-therapy [27]. Gao et al. specifically reviewed studies on symptom change before and after lockdown, including one covering the reopening phase. They found that lockdown worsened ED symptoms and related anxiety and depression, but these symptoms returned to baseline levels during the reopening phase. In addition, Sonne et al. [28] report a transient rise in diagnosed ED among Danish youth and young adults during the COVID-19 pandemic, followed by a subsequent return to pre-pandemic rates.

Stress, driven by the uncertainty of the COVID-19 pandemic, may have contributed to worsening of ED symptoms [20, 24, 26, 29–32], especially in countries where strict regulations were applied. Termorshuizen [33] discovered that at the onset of the pandemic, a relatively higher proportion of individuals in the Netherlands and the United States reported a perceived exacerbation of ED symptoms compared to Sweden. This disparity could be attributed to Sweden's lack of strict regulations aimed at curbing the virus's spread. Consequently, the stricter regulations implemented in the US and the Netherlands might have prompted a more pronounced initial reaction among those with EDs compared to Sweden. Termorshuizen supported this hypothesis with longitudinal analysis data from 15 countries, including Sweden, which revealed that increased policy stringency correlated with poorer mental health outcomes in the general population [34].

In summary, while rapid reviews have been crucial for informing short-term policies, there is a growing need to understand the pandemic longer-term effects and the fluctuation of symptoms across multiple lockdown and reopening periods. Unfortunately, longitudinal studies examining the pandemic’s impact on EDs are limited and often cover short timespans, such as a single lockdown period. Additionally, the role of COVID-19-related stress has yet to be considered. Especially in countries with strict regulations, COVID-19-related stress may have negatively affected individuals suffering from an ED. Addressing these literature gaps, the present study aimed to investigate the pandemic’s impact on the development and the persistence of ED symptoms in adults undergoing treatment. To this end, we conducted monthly assessments of ED symptoms over a 2-year period, starting in August 2020, among a cohort of patients receiving treatment for their ED. This study investigated the following hypotheses: (1) There is a positive relationship between COVID-19-related stress and the severity of ED symptoms, (2) the severity of ED symptoms will be higher during the lockdown periods compared to the reopening periods, (3) COVID-19-related stress varies across different periods, with higher levels observed during lockdowns, and (4) the relationship between period (lockdown versus reopening) and ED severity is mediated by COVID-19-related stress, indicating that ED symptoms are higher during lockdown than during reopening periods, which may be explained by more stress during lockdown periods.

## Methods

This study did not fall under the scope of the Dutch Medical Research Involving Human Subjects Act (WMO) and therefore did not require ethical approval under this regulation. The study was reviewed and approved by the Scientific Research Committee of GGZ Oost Brabant. This committee confirmed that the study adhered to the criteria for responsible research practices as outlined by both our institution’s ethical guidelines and national regulations. Informed consent was obtained from all participants prior to their involvement in the study. No compensation was provided to participants.

### Study design

The current study was an observational, longitudinal study including outpatients with a diagnosed ED. Data collection occurred between August 2020 and August 2022.

Every 4 weeks on Monday questionnaires assessing COVID-19-related stress and ED psychopathology were sent out by e-mail manually by the principal investigator to all patients receiving treatment for their ED. One reminder was sent at the end of the week if an individual did not respond. Beyond ED diagnosis, no additional participant information was collected, due to privacy considerations and the need to minimize participant burden in a small and specialized clinical sample, where the combination of diagnostic subgroups and treatment timing could increase the risk of identifiability. Once treatment stopped, patients stopped receiving the questionnaires, as the primary reason for completing the questionnaire was evaluating who was at risk for developing COVID and COVID-related symptom deterioration and stress. Monitoring patients' health was important for our patients' and therapists’ physical and mental health, as well as to inform our treatment policy. The study stopped as soon as the COVID pandemic was over. Qualtrics was used for data collection. Reasons for drop-out and missing data were not recorded.

### Participants

The sample consisted of outpatients from the Center of Eating Disorders, Mental Health Center Region Oost Brabant (Helmond, the Netherlands). The Centre for Eating Disorders provides (supra-)regional care to patients with severe EDs. The treatment center functions as a specialized facility serving patients who have not benefited from (or will likely not benefit from) less intensive, treatment settings (e.g., individual therapy, primary mental health care, other secondary health care in the region and country). The center offers highly specialized care through a comprehensive multimodal treatment program, designed to normalize eating behavior, stabilize or restore body weight, and reduce or eliminate compensatory behaviors. Our therapeutic approach is eclectic, integrating evidence-based methods such as Cognitive Behavior Therapy–Enhanced (CBT-E), Specialist Supportive Clinical Management (SSCM), and the Maudsley Model of Anorexia Nervosa Treatment for Adults (MANTRA), primarily delivered in group formats. Patients receive treatment two to five full days a week, depending on the severity of their ED. During the pandemic, depending on the severity of the lockdown requirements and the number of COVID-19 cases in the patients groups and teams, patients received blended face-to-face and online treatment. Face-to-face treatment was provided when possible.

### Measures

#### COVID-19 peritraumatic distress index

The COVID-19 Peritraumatic Distress Index [5] assesses peritraumatic psychological stress symptoms related to the COVID-19 pandemic. Participants indicated the extent to which 24 items applied to them over the past week using a 5-point Likert scale (0 = never to 4 = mostly). Example items include “I avoid watching COVID-19 news, since I am scared to do so”, and “During this COVID-19 period, I often feel dizzy or have back pain and chest distress”. The CPDI has an excellent reliability [5, 35], and has established content validity [5, 36]. However, a validated Dutch version of the CPDI does not exist. Therefore, the authors translated the questionnaire from English to Dutch without using a formal back-translation method. The internal consistency of the CPDI in this study was deemed acceptable at the initial measurements of each period (α = 0.85–0.94).

#### Eating disorder examination questionnaire

The Dutch version of the Eating Disorder Examination Questionnaire (EDE-Q; [37] assess ED psychopathology. The EDE-Q, encompassing both the total score and subscales, demonstrates good test–retest reliability and internal consistency, with Cronbach’s alpha values ranging from 0.70 to 0.93 [38–43]. Furthermore, the EDE-Q exhibits strong convergent and divergent validity [44–47]. For this study, the internal consistency of the total EDE-Q was excellent at the first measurement of each period (α = 0.93–0.95).

#### Statistical analysis

**Fig. 1:**
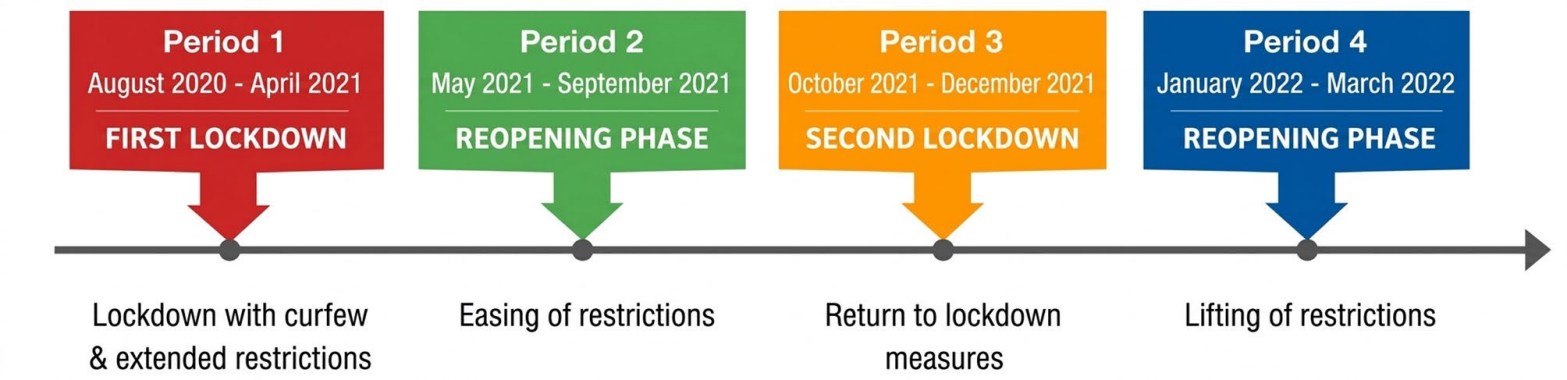
Timeline of Lockdown and Reopening Periods during the COVID-19 Pandemic in the Netherlands

Linear Mixed Models were used for the analysis. We applied Maximum Likelihood estimation to all models. The study period comprised two lockdown periods and two reopening periods (see Fig. 1).

**Fig. 2:**
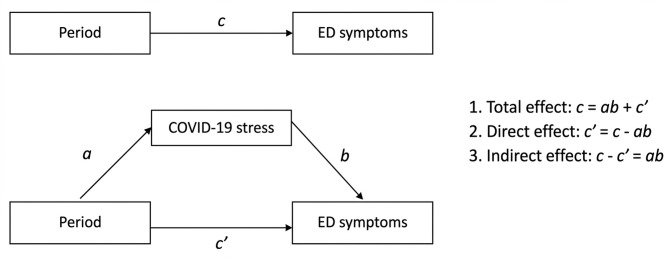
Mediation Model. The mediation model with three main pathways. First, the total effect (c) represents the overall relationship between Period and ED symptoms, including both direct and indirect effects. Second, the direct effect (c') is the effect of Period on ED symptoms after accounting for the mediation effect of COVID-19-related stress. Third, the indirect effect (ab) is the effect of Period on ED symptoms through the mediator, COVID-19-related stress

Due to the anticipated rise and decline of EDE-Q and COVID-19-related stress during and after the lockdown, we combined both periods of lockdown as well as both periods of reopening. Period (lockdown vs. reopening) was modeled as a fixed effect, and random slopes for Period were included to allow the effect of Period to vary between participants. Calendar time was not explicitly included as a predictor, and gradual or nonlinear symptom trajectories over time were not modeled. To explore the role of COVID-19-related stress as a mediator between period and ED symptoms, we implemented the four-step analysis as proposed by Baron and Kenny [48]; see Fig. 2. According to this methodology, COVID-19-related stress is confirmed as a mediator if the direct effect (c’) is significantly less than the total effect (c). Full mediation is indicated when the direct effect (c’) is non-significant and approaches zero, indicating that the mediator fully accounts for the relationship between Period and EDE-Q.

Model I was created with the EDE-Q total score as the dependent variable and Period (lockdown versus reopening) as the independent variable, including a random intercept and a random slope for Period at the patient level (path c). Model II was created with COVID-19-related stress as the dependent variable and Period as the independent variable, including a random intercept at the patient level (path a). Model III was created with the EDE-Q total score as the dependent variable and COVID-19-related stress as the independent variable, including a random intercept and a random slope for Period at the patient level (path b). Model IV was created with the EDE-Q total score as the dependent variable and both COVID-19-related stress and Period as independent variables, including a random intercept and a random slope for Period at the patient level. For each linear mixed-effects model, the intraclass correlation coefficient (ICC) was calculated to estimate the proportion of variance in ED symptoms attributable to between-person differences versus within-person fluctuations over time. All models were subjected to checks for normality of residuals, homoscedasticity, and multicollinearity. Where residuals did not demonstrate normal distribution, transformations of the dependent variables were considered. Descriptive statistics were utilized for data summarization, incorporating means and standard deviations, medians and interquartile ranges, as well as frequencies and percentages, adapted according to the nature of each variable under consideration. Analyses were performed in R, version 4.3.1, using packages lme4 and lmerTest.

## Results

### Patient characteristics

One hundred and eight patients participated. Table 1 shows an overview of the samples’ clinical diagnoses.

**Table 1:** Patients’ baseline characteristics: clinical diagnosis

Diagnosis	EDEQ_MEAN		CPDI_SUM
	N (%)	Mean (SD)	Mean (SD)
Anorexia nervosa	42 (38.9)	3.56 (1.43)	23.14 (12.94)
Bulimia nervosa	35 (32.4)	3.13 (1.53)	20.91 (16.41)
Other^a^	14 (13.0)	3.96 (1.08)	21.85 (17.79)
Binge eating disorder	7 (6.5)	2.95 (0.96)	20.14 (21.39)
Other specified feeding- or eating disorder	5 (4.6)	3.29 (1.23)	21.40 (16.46)
Unspecified feeding- or eating disorder	5 (4.6)	3.24 (0.88)	21.40 (11.99)
Total	108 (100)	3.40 (1.38)	21.90 (15.15)

This table represents main diagnoses conform the DSM 5 criteria. All disorders are presented separately

^a^Participants diagnosed with the diagnosis ‘other’ had a primary diagnosis outside the ED spectrum but received supplemental treatment for ED as a secondary diagnosis

### Hypotheses testing

**Fig. 3:**
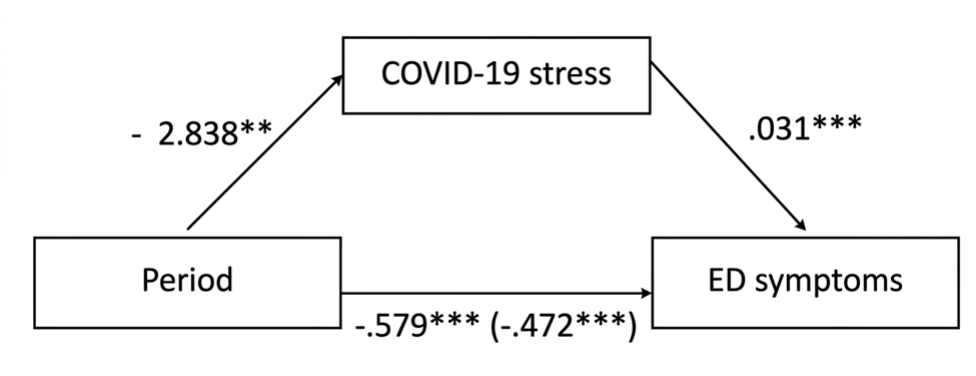
Mediation Model of the Relationship Between ‘Period’ and ‘Eating Disorder Symptoms’ Through ‘COVID-19-related stress’. Unstandardized estimated regression coefficients for the relationship between period and eating disorder symptoms as mediated by COVID-19-related stress. The unstandardized estimated regression coefficient between period and eating disorder symptoms, controlling for COVID-19-related stress, is in parentheses. ^**^p < 0.01. ^***^p < 0.001

A negative and significant total effect between period and ED symptoms was observed, with the symptoms being lower during the reopening periods compared to the lockdown periods (Model I), b = − 0.579, SE = 0.137, p < 0.001 (i.e., path c in Fig. 3). The intraclass correlation coefficient (ICC) for ED symptoms in Model I was 0.77, indicating that 77% of the total variance in ED symptoms could be attributed to differences between participants, with the remaining 23% representing fluctuations within individuals over time. Secondly, it was found that during lockdown periods, patients experienced more COVID-19-related stress than during the reopening periods (Model II), b = − 2.838, SE = 1.003, p = 0.005 (i.e., path a in Fig. 3). In Model II, the ICC for COVID-19-related stress was 0.79, suggesting that most of the variability occurred between participants. Thirdly, a significant positive association between COVID-19-related stress and ED symptoms was observed (Model III), b = 0.031, SE = 0.0053, p < 0.001 (i.e., path b in Fig. 3). Even after accounting for COVID-related stress in Model III, the ICC for ED symptoms remained around 0.76, indicating that the majority of the variance in ED symptoms was still attributable to differences between individuals. Finally, a significant negative direct effect of period on ED symptoms was noted, where, controlling for COVID-19-related stress, patients experienced fewer ED symptoms during the reopening periods compared to the lockdown periods (Model IV), b = − 0.472, SE = 0.132, p < 0.001, (i.e., path c’ in Fig. 3). Similarly, the ICC for ED symptoms in Model IV was approximately 0.76, maintaining the trend of a substantial portion of variance being between participants. The indirect effect (*ab)* of period on ED symptoms was − 0.088, p = 0.01 (Sobel Test), indicating partial mediation.

## Discussion

The aim of this study was to investigate the pandemic’s impact on the development and the persistence of ED symptoms in adults undergoing treatment for their ED by examining the severity of ED symptoms during periods of lockdown compared to reopening periods. Additionally, we assessed whether COVID-19-related stress mediated this relationship. Previous studies on this topic revealed mixed outcomes and longitudinal studies examining the pandemic’s impact on EDs are limited; most did not cover multiple periods of lockdown and reopening, and also did not include COVID-19-related stress.

Consistent with our hypotheses, patients experienced more ED symptoms during lockdown periods compared to reopening periods. COVID-19-related stress was also higher during lockdown periods compared to reopening periods, and higher levels of COVID-19-related stress were related to more severe ED symptoms. The mediation analysis showed that ED symptoms were higher during lockdown than during reopening periods, partly due to more COVID-19-related stress during lockdown periods.

Our findings are in line with the majority of several reviews and meta-analyses (e.g., 15, 17–22), which found a positive relation between COVID-19-related stress and ED severity. Our findings are also in line with Schneider et al. [23], who acknowledged a general worsening of symptoms, although not all individuals were adversely affected, as well as that these symptoms returned to baseline levels during the transition from lockdown to reopening. Similarly, Gao et al. [16] found that lockdown worsened ED symptoms compared to reopening periods. In addition, our results align with Phillipou et al. [49], who reported that individuals with an ED history had higher mental health symptoms than those without, with both groups showing worsening of depressive symptoms, restrictive eating, and quality of life during a lockdown. These findings highlight the vulnerability of ED populations to pandemic-related stress.

Secondly, we found that patients experienced fewer ED symptoms during reopening periods, compared to lockdown periods, and that this was partly due to less COVID-19-related stress during reopening periods. Higher levels of stress during lockdown periods are associated with uncertainties as a result of the strict measures to restrict spreading of the virus especially in countries with strict regulations [11, 20, 24, 26, 29–32].

This study had several important strengths. To start, this study examined the longitudinal effect of COVID-19-related stress on ED symptoms, which made it possible to directly compare lockdown periods to reopening periods. Covering a period of 2 years, the current study is the longest of its kind. Also, including a measure of COVID-19-related stress, we were able to investigate the role of COVID-19-related stress in the worsening of ED symptoms during lockdown. In this manner, this study provides an expansive perspective on a dynamic area of research.

When interpreting the present findings, it is important to consider several limitations. First, this study relied on self-reported data, which increases the potential for response bias, including both under-reporting (due to social desirability or lack of awareness of ED symptoms) and over-reporting (due to heightened distress). Second, the CPDI, which assesses COVID-19-related stress, was translated into Dutch by the authors themselves without using a formal back-translation method. Furthermore, this study was based on self-selection, and it is unclear whether patients who did not agree to participate, differed in their clinical conditions from those included in the study, which may introduce selection bias. Another limitation is the sparse demographic information available; beyond ED diagnosis, no additional data on age, socioeconomic status, living situation, or other potentially relevant factors were collected. This constraint reflects privacy considerations and the need to minimize participant burden in a small and specialized clinical sample, where the combination of diagnostic subgroups and treatment timing could increase the risk of identifiability. The absence of broader demographic information limits the ability to examine individual difference factors that may shape vulnerability or resilience to pandemic-related stress. Lastly, because calendar time and individual symptom trajectories were not explicitly modeled, the present study could not examine heterogeneity in the timing or shape of responses to pandemic-related stress.

Future research is needed to obtain a more comprehensive understanding of protective and risk factors that may influence the long-term consequences of the pandemic for people with EDs, particularly in explaining why some individuals appear more adversely affected than others. Prior research (e.g., 23) suggests that such individual differences may be related to clinical factors (e.g., diagnosis, illness severity, BMI, psychological characteristics (e.g., emotion regulation strategies, intolerance of uncertainty), and contextual or social factors (e.g. social support, time spent online). Schneider et al. mention these factors as potential risk or protective factors. In addition, demographic characteristics such as age and gender, as well as treatment-related factors (e.g., continuity of care or access to face-to-face treatment during lockdowns), may contribute to differential vulnerability or resilience. Future longitudinal studies explicitly modeling individual trajectories could help disentangle how these factors interact with prolonged stress exposure, thereby providing a more nuanced understanding of heterogeneity in responses to large-scale public health crises and informing more targeted preventive and therapeutic interventions for vulnerable groups.

## Conclusion

This study examined how COVID-19-related stress affects ED symptoms, comparing lockdown and reopening periods over a period of 2 years. The results showed that COVID-19-related stress partially and negatively explained the relationship between lockdown and reopening periods and ED symptoms. We also found that both ED symptoms and COVID-19-related stress increased during the lockdown, with a positive association between COVID-19-related stress and the severity of ED symptoms. Altogether, our findings suggest that ED symptoms worsen partly due to increased COVID-19-related stress during lockdown periods, in the Netherlands—a country where strict regulations were applied. This study adds unique information to our understanding of the longitudinal impact of stress during a pandemic in patients with an ED. These findings can inform strategies for addressing the mental health and ED challenges faced during future pandemics, helping to better prepare for similar global crises and their effects on at-risk populations.

## Data Availability

The data that support the findings of this study are available from the corresponding author upon reasonable request.
